# Highly Efficient Enzymatic Preparation of Daidzein in Deep Eutectic Solvents

**DOI:** 10.3390/molecules22010186

**Published:** 2017-01-22

**Authors:** Qi-Bin Cheng, Li-Wei Zhang

**Affiliations:** 1 Institute of Molecular Science, Key Laboratory of Chemical Biology and Molecular Engineering of Ministry of Education, Shanxi University, Taiyuan 030006, China; qbcheng1992@163.com; 2 College of Chemistry and Chemical Engineering, Shanxi University, Taiyuan 030006, China

**Keywords:** deep eutectic solvents, daidzein, enzymatic hydrolysis, response surface methodology, optimization

## Abstract

Daidzein, which is scarce in nature, has gained significant attention due to its superior biological activity and bioavailability compared with daidzin. So far, it has been widely used in the medicine and health care products industries. The enzymatic approach for the preparation of daidzein has prevailed, benefitted by its high efficiency and eco-friendly nature. Our present research aimed at providing a preparation method of daidzein by enzymatic hydrolysis of daidzin in a new “green” reaction medium-deep eutectic solvents (DESs). Herein, the DESs were screened via evaluating enzyme activity, enzyme stability and the substrate solubility, and the DES (ChCl/EG 2:1, 30 vol %) was believed to be the most appropriate co-solvent to improve the bioconversion efficiency. Based on the yield of daidzein, response surface methodology (RSM) was employed to model and optimize the reaction parameters. Under these optimum process conditions, the maximum yield of 97.53% was achieved and the purity of daidzein crude product reached more than 70%, which is more efficient than conversions in DESs-free buffer. Importantly, it has been shown that DESs medium could be reused for six batches of the process with a final conversion of above 50%. The results indicated that this procedure could be considered a mild, environmentally friendly, highly efficient approach to the economical production of daidzein, with a simple operation process and without any harmful reagents being involved.

## 1. Introduction

Soybean isoflavones is a class of biologically active secondary metabolites which are formed in the soybean growth and belong to the group of flavonoids. Their structure and function are similar to human estrogen, which could play a role in the prevention of osteoporosis, cancer, cardiovascular diseases and postmenopausal syndromes [1,2,3]. The existing research has reported that there are 12 kinds of soybean isoflavone isomers isolated and divided into free aglycones and glucosides, including daidzein, genistein, glycitein and in the form of acetyl-, malonyl- and β-glycosides, respectively. [4]. Williamson, Heubi and Kikuchi, et al. [5,6,7] found that the aglycones form of soybean isoflavone could be efficiently absorbed by the small intestine only after glycosyl is removed by the hydrolysis of glycosides. Therefore, isoflavone aglycones are superior to isoflavone glycosides in their various bioactivities and effective absorption [8,9,10,11]. Due to its low content in the soy dry extract, high-efficiency conversion of isoflavone glycosides into aglycones has gained enough attention and become a focus in the food chemistry-related fields.

Although a variety of hydrolysis methods of soybean isoflavone glycosides are available [12,13], such as acidic and alkali catalytic hydrolysis, acetolysis and Smith degradation, etc., they might bring some adverse effect on the stability of daidzein in strong acid hydrolysis and their products would more be prone to degradation in a strong alkaline environment. The mild enzymatic hydrolytic process using isolated β-d-glucosidase has been proved to be effective and preponderant [14,15,16,17]. Due to poor solubility or affinity of the substrate in the buffer [18], we will attempt to find a new kind of green and sustainable solvent with high solubility and affinity to ensure the process is simpler and more efficient.

In 2003, Abbott, et al. [19] found that the liquid formed by choline chloride and amide compounds had special physical properties, and put forward the concept of deep eutectic mixture for the first time. Subsequently, the deep eutectic mixture was gradually developed into a new type of green solvent-deep eutectic solvents (DESs). DESs can be easily prepared by thermal mixing with an ammonium salt (such as choline chloride) and a hydrogen-bond donor (HBD, such as urea and glycerol) that are capable of self-association at a specified stoichiometric ratio [20,21]. The resulting DESs had a higher viscosity and lower melting point than that of each individual component [19,21]. Due to the strong interaction between the HBD and the salt anion through the extensive hydrogen bond network, DESs possess unique advantages, such as modest, eco-friendly, high atom economy, non-toxicity, high biodegradability and enzyme compatibility. More importantly, the nature of the DES’s performance would be different by changing composition or mole ratio to exhibit unique designability [22,23,24]. Driven by these excellent properties and advantages, DESs have become promising and attractive as new types of nonaqueous solvent/co-solvent for various emerging applications including biocatalysis [25,26,27]. To process the benefit of the fairish biocompatibility and suitable properties, DESs have been successfully applied to some specific enzymatic reactions, producing a favorable influence on the activity and stability of the enzyme [28,29,30]. For some stereoselective reactions, the selectivity of reaction is also greatly improved [25]. In the enzymatic hydrolysis assays, DESs have been proven to be a good solvent candidate for the hydrolase-catalyzed biotransformations of styrene oxide and p-nitrophenyl acetate [31]. Although there are only a few similar studies, these results have suggested that DESs deserve consideration as a potential green solvent for enzymatic processes. Owing to these remarkable advantages, meanwhile, considering the above problems, DESs were expected as a type of appropriate medium to be applied to the enzymatic hydrolysis of soybean isoflavone glycosides.

In this study, the model reaction of hydrolying daidzin to generate daidzein by β-d-glucosidase in DES-containing aqueous solution was evaluated. First, a total of 16 DESs of choline chloride (ChCl) combined with five hydrogen-bond donors (HBDs) (i.e., urea (U), propylene glycol (P), glycerol (G), ethyleneglycol (EG) and glucose (Glu)) at the molar ratios (2:1, 1:1, 1:2, 1:3) were prepared and tested. Second, equilibrium solubility of daidzin in several representative DESs was studied, and the influence of DESs (DESs as a co-solvent in aqueous solvent) on enzyme activity and stability of β-d-glucosidase was explored. Some enzymatic parameters were also determined. Furthermore, in the enzymatic reaction, the crude extract containing 60% daidzin was employed as the substrate for preparation of daidzein, and the effects of different parameters on the enzymatic reaction were optimized by response surface methodology (RSM), and the optimal process conditions were confirmed. The encouraging results not only provided a preferable enzymatic reaction in the new medium, but the productivity and products purity was enhanced markedly in a low enzyme dosage. The fine performance suggests that DESs might be a competitive candidate for the medium of enzymatic reactions, along with the fact that there are no harmful reagents involved and efficiency is high.

## 2. Results and Discussion

### 2.1. The Screening of Optimum DES for the Enzymatic Reaction Based on Activity and Stability

**Figure 1:**
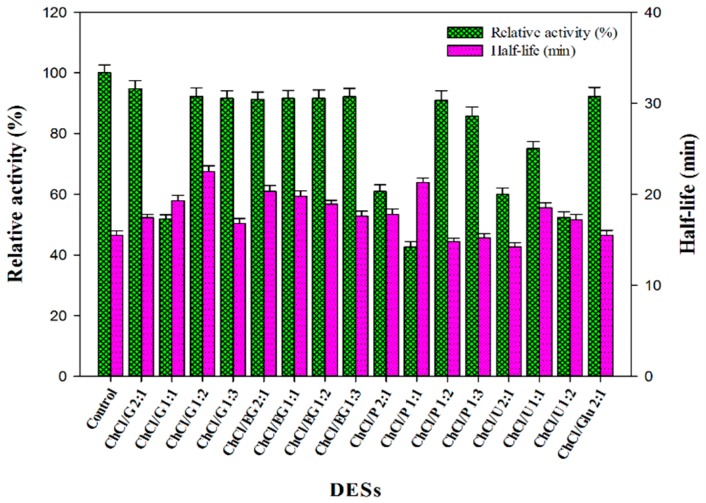
Activity and thermal-stability of β-d-glucosidase in phosphate buffer (0.1 M, pH 5.8) containing different DESs (20 vol %). The relative activities (%) refer to the percentages of the initial reaction rates obtained by the enzyme in the deep eutectic solvents (DES)-containing aqueous solutions relative to the one obtained in the pure buffer. The half-life (min) of the enzyme were obtained at 65 °C.

The 16 DESs were screened for their influence on the catalytic performance of β-d-glucosidase in aqueous solutions for the first time. The various DESs with ChCl as hydrogen bond receptor and the HBD included polyols, carbohydrate and amide, and the molar ratio between ChCl/HBDs were confirmed at 2:1, 1:1, 1:2 and 1:3, respectively. Using the enzymatic activity in the phosphate buffer (pH 5.8) as 100%, the relative activity and stability parameters were measured in the presence of different DESs-containing aqueous solutions (20 vol %). As can be seen in Figure 1, the regularity of enzyme action could be shown intuitively, both the activity and stability of β-d-glucosidase in different DESs-containing aqueous solutions were varied, depending on the choice of the HBDs and the molar ratio between ChCl/HBDs. In terms of the HBDs, polyols was superior to amide in maintaining the enzymatic activity. In polyols-based DESs, the enzymatic activity showed similar results at the molar ratio of 2:1, 1:2 and 1:3, while it decreased markedly at 1:1 molar ratio. This might be the reason for the weak hydrogen bonding interaction between ChCl and polyols at lower molar ratio, thus increasing the rigidity of enzyme structure and damaging the activity center. However, the enzyme performance was not completely consistent compared to that in urea (U)-based DES, in terms of the influence of HBDs, the results may be attributed to the different medium environment and strength of hydrogen bond network. Additionally, the inverse effects were reflected on the thermal stability, when ChCl and HBDs had a molar ratio of 1:1, the half-life of the enzyme was significantly longer compared to that at other molar ratios. Comprehensively considering the above results, the four candidate representative DESs (i.e., ChCl/G 2:1; ChCl/G 1:2; ChCl/EG 2:1 and ChCl/Glu 2:1) were employed to assess the storage stability of β-d-glucosidase, and the results were displayed in Figure S1.

In terms of the overall impact of DESs on the enzyme performance, the decreasing extent of enzymatic activity remained within an acceptable level that the relative activity of the enzyme was maintained above 90%. To our satisfaction, the β-d-glucosidase exhibited higher thermal and storage stability in most DESs-containing aqueous solutions compared with that in DESs-free solution. The stability of the enzyme is a key parameter in the practical application process [32], especially in the enzymatic reaction with a long duration. Thereby, it is a crucial adjective index of evaluation in the screening test.

### 2.2. The Influence of Different DESs as Additives on Enzymatic Activity and Stability

**Figure 2:**
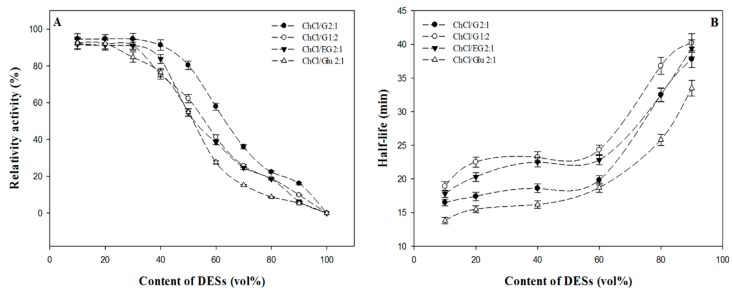
Variation of β-d-glucosidase activity (**A**) and half-life (**B**) in the four typical DESs (ChCl/G 2:1; ChCl/G 1:2; ChCl/EG 2:1 and ChCl/Glu 2:1) with different concentration. The half-life (min) of the enzyme were obtained at 65 °C.

It is important to take full advantage of the DESs-containing aqueous solutions benefits, which has presented a comparable or even superior performance for some specific enzymatic reactions. In this step, a detailed assessment regarding the enzymatic activity and stability in the presence of different DESs-containing aqueous solutions (DESs content of 10–90 vol %) was conducted. The enzyme activity and stability were used to evaluate the influence of different concentrations of DESs on β-d-glucosidase performance. As shown in Figure 2A, for the four typical tested DESs, a noticeable increase in the DESs concentration caused the enzyme to be less active but much more stable. When the buffer content was below 40 vol %, the amplitude of variation of enzymatic activity remained at an acceptable level, but a sharp fall appeared once this critical concentration was exceeded. In allusion to the phenomenon, this may not be attributed to protein denaturation, but rather to destabilization of enzyme-substrate or reaction intermediate complexes [28,33,34,35]. Due to the effect of new hydrogen bonding formed between water and ChCl, some researchers [23,36,37] have proved that the strong hydrogen-bonding network might be damaged with the large addition of water. It is possible that in aqueous solution the strong hydrogen-bonding network extensively present in the DESs may still at least partially remain, and still play an important role in their physicochemical properties and their applications.

In non-aqueous enzymatic catalysis [38,39], a certain amount of water is essential to ensure the reaction activated and proceed, which could contribute to the maintenance of enzyme structural flexibility and necessary conformation. In the meantime, the viscosities of DESs were greatly reduced, and the substrate was released from the hydrogen-bonding network, which was conducive to the combination of the substrate with the enzyme active center and lead to a significant increase in the enzymatic efficiency. These results highlighted the benefits of water in terms of regulating properties of DESs. In this regard, Dai et al. [37] have done a series of detailed researches on tailoring properties of DESs with water to facilitate their applications. Figure 2B displayed that the stability of β-d-glucosidase increased dramatically as the water content decreased. By investigating the thermostability and storage stability of the enzyme, we found that it had a longer half-life in DESs-containing aqueous solutions compared with that in pure buffer, which agreed with the previous study [40]. This might be related to the DES’s strong hydrogen-bonding network by acting on the structure of an enzyme. The results mean that defining the equilibrium point of enzymatic activity and stability would be essential for different types of reactions.

Regarding the research of non-aqueous enzymology, the thermodynamic water activity (a_w_) is a measurement index of the effective concentration of water in a mixed system. Theoretically speaking, the hydrolysis reaction will be properly impaired by adding DESs, but this phenomenon did not occur, which could be explained by the strong association of the hydrogen-bond network formed between the substrate and DESs [37,41]. It is encouraging that β-d-glucosidase could still maintain a high level in catalytic ability owing to the integrality of the space structure and active center. On the other hand, it indicated that the “essential water” of β-d-glucosidase was not deprived in DESs with 30 vol %.

Not only did DESs-containing aqueous medium affect the structure of the enzyme, but could change some physical parameters (surface tension, viscosity, etc.) to have a deep-seated effect on the enzymatic reaction [31,42]. An increase in solvent viscosity could augment the diffusion resistance of substrates, thus enhancing the mass transfer limitations and impairing the interactions between enzyme particles and substrates. Therefore, in order to explore the mechanism more objectively and scientifically, we need to take the specific reaction and the type of DESs into full consideration.

### 2.3. The Parameters of Enzymology Properties and Enzymatic Reaction Kinetics in DESs

**Figure 3:**
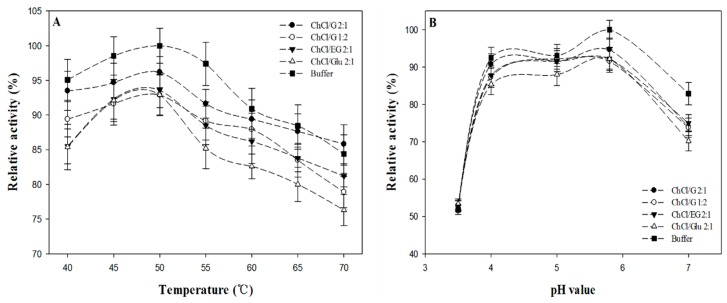
Effect of temperature (°C) (**A**) and pH value (**B**) on the activity of β-d-glucosidase in the four typical DESs (ChCl/G 2:1; ChCl/G 1:2; ChCl/EG 2:1 and ChCl/Glu 2:1).

It is well known that pH and temperature of the environment significantly affect the catalytic activity of the involved enzymes [41]. The optimum pH and temperature could make the enzymatic performance more effective. Hence, we determined the influence of pH and temperature on the enzyme activity in four typical DESs (i.e., ChCl/G 2:1; ChCl/G 1:2; ChCl/EG 2:1 and ChCl/Glu 2:1, 20 vol %). The effect of DESs on the optimum temperature and pH of β-d-glucosidase were preliminarily investigated and the results were described in Figure 3. In the enzymology properties curve, we can find that optimal temperature was 50 °C and pH value was 5.0–5.8, which has no obvious alterations with the addition of DESs compared to that in pure buffer. This may be attributed to the fact that the structure of the enzyme has not been significantly damaged and the structure integrality is still maintained in the co-solvent. In the external medium, the optimum pH of the environment usually depended on the pH value of the buffer before freeze-drying, which is defined as “pH-imprinting” [43].

In our previous work, the enzymatic Kinetics curves of β-d-glucosidase in ChCl/EG 2:1-containing aqueous solutions and phosphate buffer (pH 5.8) were also determined, and the classical Michaelis constant (K_m_) value and maximum reaction rate (V_max_) were calculated via Lineweaver-Burk plots, as observed. The results showed that V_max_ of the enzymes in the DESs-free buffer solution was 2.5 μmol·min^−1^·mg^−1^, and the K_m_ value was 1.8 mM; in ChCl/EG 2:1 30 vol % solution system, the reaction V_max_ dropped to 1.65 μmol·min^−1^·mg^−1^, and the K_m_ value dived to 0.85 mM. The results indicated that the affinity between the enzyme and DESs-containing aqueous solutions was higher than those between the enzyme and DESs-free buffer. Moreover, the DESs’ strong hydrogen-bond network is more beneficial in the role of induced-fit between the enzyme and the substrate. At the same time, the viscosity of DESs played a significantly important role in the enzyme kinetics by affecting the maximum reaction rate V_max_. Therefore, it was not surprising that the V_max_ in DES was inferior to that in DESs-free buffer. The results were consistent with those published by Sheng-Li Yang et al. [41] and Bubalo et al. [42], who reported that the higher catalytic activity of the enzymes was observed in DESs with lower viscosity.

### 2.4. Equilibrium Solubility of Daidzin in Different DESs

**Figure 4:**
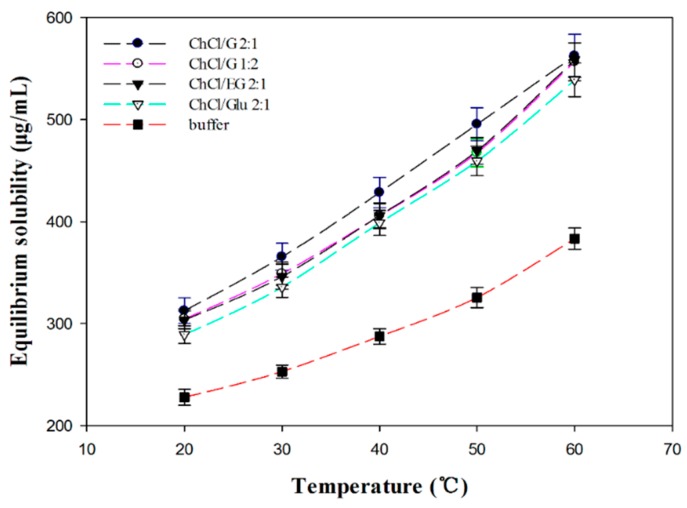
The equilibrium solubility of daidzin in the four typical DESs (ChCl/G 2:1; ChCl/G 1:2; ChCl/EG 2:1 and ChCl/Glu 2:1) at different temperatures (°C).

The tremendous capacity of DESs to dissolve polar substrates plays an important role in their availability for enzymatic reactions. The solubilities of daidzin in different DESs-containing solution (DESs-free buffer solution, ChCl/G 2:1, ChCl/G 1:2, ChCl/EG 2:1 and ChCl/Glu 2:1, 30 vol %) at various temperatures were presented in Figure 4. As can be observed, the solubility of daidzin in all the solvents increased obviously with the enhancement of the temperature, and the saturation concentration in ChCl/EG 2:1 reach to 556.7 μg/mL at 60 °C, while only 383.2 μg/mL in DESs-free buffer. The solubility showed dramatic enhancement (up to 1.5-folds) in ChCl/EG 2:1 compared with that in DESs-free buffer although the polarity of water was relatively higher. Overall, the solubility of daidzin in the screened typical DESs showed a high similarity and the variation tendencies with temperature were similar to each other. There are still some subtle differences in the solubility of daidzin in different DESs, the reasonable inference for the above phenomenon may be due to the differences in hydrogen bonding interaction although these polyols-based DESs possessed similar polarity. The results suggested that not only the solvent polarity but also other factors such as intermolecular interaction, and especially, the ability to form a hydrogen bond between the solvent and the solute as well as the physical properties of the solvent (e.g., viscosity and surface tension), could affect the solubility of the solute [44].

### 2.5. The Establishment of the Optimum Process Conditions

In order to define the effect of various independent variables on the response of hydrolysis, single-factor experiments were required to select the main variables and levels for the Box-Behnken statistical design (BBD). Various factors (DES content, reaction temperature, system pH, reaction duration, enzyme loading and substrate concentration) were investigated, and the results were depicted intuitively in Figure S3. Based on the observation of single-factor tests, several factors had been ascertained to affect the hydrolysis efficiency significantly, and the four critical parameters (reaction duration, reaction temperature, system pH and enzyme loading) were screened as the independent variables.

The main aim of the optimization process was to maximize the response value in the hydrolysis to obtain the most appropriate conditions for maximum conversion yield of daidzein. The range of operating variables time duration (min), reaction temperature (°C), system pH and enzyme loading (U) were decided via above single factor test results as listed in Table 1 in their coded levels. The BBD design yielded 29 different formulations expressed in natural units, for which the responses were summarized in Table S1. The model adequacy checking was implemented on the experimental data to define whether the approaching model would give deprived or misleading results. The experimental data could be fitted with a quadratic model by corresponding regression analysis, and a second order polynomial quadratic equation model could be obtained as follows:

*Y* = 87.34 + 9.93*A* − 17.60*B* + 24.84*C* + 15.13*D* − 0.20*AB* + 3.57*AC* + 0.38*AD* − 10.98*BC* − 1.62*BD* + 5.67*CD* − 9.14*A*^2^ − 16.52*B*^2^ − 38.78*C*^2^ − 11.35*D*^2^ (1)

The evaluation of the polynomial model equation significance was carried out by Fisher’s statistical test for the analysis of variance (ANOVA; Table 2). The *F*-value of 62.75 for response suggested that this model was statistically significant; the *p*-value less than 0.01% implied no more than a 0.01% chance that insignificance of “Model *F*-Value” could occur due to noise. The “Lack of Fit *F*-value” of 1.87 implied the Lack of Fit was not significant relative to the pure error. There was a 28.58% chance that a “Lack of Fit *F*-value” this large could occur due to noise. The coefficient of determination (Pred*R*^2^) was 0.9212, indicating that this model could explain 92.12% of the total variations. The adjusted coefficient of determination (Adj *R*^2^) was 0.9686, suggesting a good fitting of the regression model. For an approved model, “Adeq precision” larger than 4 is desirable, and the high value of 23.789 in this model indicated an adequate signal, implying that this model was competent for the design optimization.

**Figure 5:**
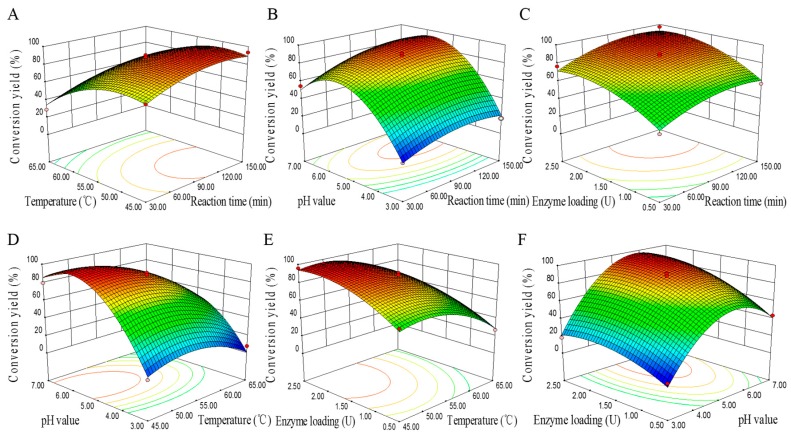
Response surface 3D plots showing the effect of (**A**) reaction time and **t**emperature; (**B**) reaction time and system pH; (**C**) reaction time and enzyme loading; (**D**) temperature and system pH; (**E**) system pH and enzyme loading; (**F**) temperature and enzyme loading on the conversion yield of daidzein.

Given the *p*-values of each model terms, it could be determined that four linear coefficients (A, B, C and D), four quadratic coefficients (A^2^, B^2^, C^2^ and D^2^) and one interactive coefficients (BC) were significant, which indicated the pattern of the interactions between the tested variables (*p* < 0.01). As displayed in Figure 5, three-dimensional surface plots provided visual interpretation of the relationship between response and experimental levels of each variable and the type of interactions between two test variables, in order to obtain the optimal hydrolysis conditions that would maximize the conversion yield of daidzein. According to the optimization of the regression model, the optimum conditions were as follows: temperature 53 °C, pH 5.35, enzyme loading 1.68 U, and reaction time 100.5 min. Under the fine-tuned conditions, parallel tests were repeated three times, and the actual conversion yield was up to 97.53%, which was in good accordance with the predicted value. Furthermore, this model was also validated with additional experiments under the selected random conditions while fixing the reaction time at the optimized value. Therefore, the predicted model was considered to be reliable and robust.

### 2.6. Preparation of Daidzein and Medium Reusability

A bench-scale test was employed to verify the feasibility of preparation for daidzein under the determined optimum technological conditions, and the reusability of recycled medium was also researched. In the amplifying system, the conversion yield of daidzein was still maintained at more than 95%, which indicated that the process was feasible even on a larger scale. Due to poor solubility of daidzein in ChCl/EG 2:1, the generated daidzein will be continually precipitated, which leads to the simple and convenient purification process. After repeatedly feeding six times at the large reaction setup shown above, the product was easily separated by collecting the sediment. Final product purification with established refinement procedures, the purity of daidzein crude product greater than 70% (m/m) was achieved. This was considered a satisfying result despite the low purity of the substrate that was employed, which is self-explanatory. If there was any need, we could further purify by the conventional methods (e.g., column chromatography and extraction) to obtain higher purity products.

**Figure 6:**
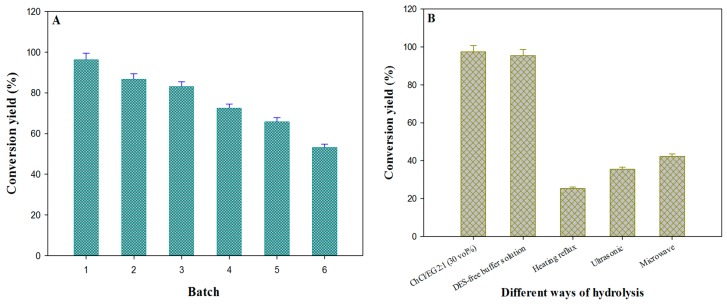
The medium reusability of recycling times (**A**) and the hydrolysis efficiency by using different ways to prepare daidzein (**B**).

As shown in Figure 6A, the results illustrated that the same medium was used six times for the hydrolysis reaction, resulting in a small decrease in enzyme activity (conversion yield from 96.4% to 65.8%) for the first five recycling cycles. Thus, high stability of the enzyme in the medium and the high concentration of the substrate are responsible for the equilibrium shift towards daidzin hydrolysis, which may have contributed to the high repeatability. Consequently, the ChCl/EG 2:1 DES medium could allow us to carry out long-time and more complex reactions that enables us to provide a high productivity.

### 2.7. The Superiority of ChCl/EG 2:1 Medium in Preparation of Daidzin

In order to verify the reliability of enzymatic process in DESs medium, acid-catalyzed hydrolysis in DESs-free buffer was employed as controlled trials, which is the most commonly-used traditional method for the preparation of daidzein. From Figure 6B, the lowest hydrolysis yield was obtained under the one-fold heating condition, while a tiny increase (reaching up to 42.3%) was seen under ultrasonic or microwave assisted conditions. Although microwave and ultrasonic energy could facilitate the reaction rate, the efficiency was still at a lower level. This may be caused by its catalytic mechanism, i.e., that the intermediate had higher activation energy and instable structure [45]. In DESs-free phosphate buffer, the hydrolysis rate of daidzin reached 95.5%, a satisfying level; but on account of the limitation of poor solubility of the substrate, it is impractical for large-scale reaction.

On the whole, the acid-hydrolysis efficiency was far less than that by enzymatic catalysis. The results come as no surprise and indicated the significant efficiency advantage provided by the enzymatic process. In other words, the enzymatic catalysis in DESs medium was fully suitable for mass production of daidzein. Because of the handy purification process and high reusability of ChCl/EG 2:1 medium, there is an absolute advantage in enzymatic preparation of daidzein compared with DESs-free buffer. Hence, the enzymatic preparation method for daidzein showed practical application value in large-scale production.

## 3. Experimental

### 3.1. Materials

Almond β-d-glucosidase was purchased from Shanghai Jianglai Chemical Co., Ltd. (Shanghai, China) with a specific activity of 20–40 U/mg. *p*-nitrophenyl-β-d-glucopyranoside (pNPG) and *p*-nitrophenol (pNP) were purchased from Chengdu Xiya Chemical Co., Ltd. (Chengdu, China). The isoflavone 60% daidzin extract was purchased from Shanghai Yiji industrial Co., Ltd. (Shanghai, China). Choline chloride (ChCl), urea (U), glycerol (G), ethylene glycol (EG), propylene glycol (P), glucose (Glu) and all other general reagents used were of analytical grade from Shanghai Yuanye Bio-Technology Co., Ltd. (Shanghai, China) and chromatogram reagents were provided by Thermo Fisher Scientific (Waltham, MA, USA). All the standard substances are provided by the Chengdu Must Bio-Technology Co., Ltd. (Chengdu, China). Agilent 1260 Infinity LC (Agilent Technologies Industries Co., Ltd., Santa Clara, CA, USA) equipped with a 1260 Quat pump VL quaternary pump, 1260 ALS autosampler, 1260 TCC column thermostat, and UV ultraviolet detector.

### 3.2. Preparation of DESs and DESs-Containing Aqueous Solutions

ChCl and each of hydrogen-bond donors (G, EG, P, U or Glu) were mixed at a molar ratio of 2:1, 1:1, 1:2 and 1:3, respectively. The mixture was performed in a round bottom flask, followed by sealing the reaction system and mixing in a magnet stirrer at 80–100 °C for 8–10 h until the solution was colourless and transparent. Following that, the resulting eutectic mixture was cooled down to room temperature and dried over P_2_O_5_ in a desiccator at room temperature for at least two weeks prior to use. The DES-contained aqueous solutions were prepared by dissolving DESs into a phosphate buffer (0.2 M Na_2_HPO_4_, 0.1 M C_6_H_8_O_7_·H_2_O, pH 5.8) with uniform mixing ensure DESs at an appropriate volume fraction.

### 3.3. Assays for β-d-Glucosidase Activity in DESs

The activity of β-d-glucosidase was determined spectrophotometrically using spectrophotometry following the hydrolysis of pNPG to produce pNP, according to the method described in reference [46] with minor modifications. The freeze-dried enzyme powders were dissolved into different DESs-containing aqueous solutions (10, 20, 30, 40, 50, 60, 70, 80, 90 and 100 vol %). The activity of the enzyme solution was kept at 2.5 U/mL (based on the activity for catalyzing pNPG hydrolysis in phosphate buffer (pH 5.8) as described below). Dissolving a proper amount of the substrate pNPG in the same DESs-containing aqueous solutions described above and produce the ultimate concentration 2.0 mmol/L, then mixed 1400 μL of substrate solution with 100 μL of enzyme solution, which both were pre-incubated in a thermostatic water bath at 45 °C for at least 10 min. Subsequently, they were mixed evenly and we allowed the reaction to continue for 20 min. After the reaction, 1.5 mL of 0.5 M Na_2_CO_3_ was added into the system terminating the enzyme reaction. For each experiment, a blank was prepared for background subtraction under the same conditions but the distilled water was used instead of substrate solution. One unit of enzyme activity was defined as the amount of enzyme catalyzing formation of 1 μmol of pNP per minute under the described conditions.

### 3.4. Tests for β-d-Glucosidase Stability in DESs

The thermal stability and storage stability were used to synthetically evaluate the enzyme stability in DESs. Thermal stability was assessed by the conventional method, that 100 μL of enzyme solution (prepared via the above method) was added to 400 μL of phosphate buffer (pH 5.8) containing different DESs with an appropriate volume fraction, incubate in a thermostatic water bath at 65 °C for 0, 15, 30, 45, 60 min. Periodically, dissolved of 1000 μL substrate solution in DESs-containing aqueous solutions with different volume fractions (10, 20, 30, 40, 50, 60, 70, 80 and 90 vol %) and mixed well gently for activity assay at 45 °C as the above method. The time-dependent loss of the activity was used to calculate the half-life of each enzyme solution in different DESs. For the determination of storage stability of β-d-glucosidase, we adopted the assay method by measuring the activity loss with a long-term storage. First, the β-d-glucosidase was dissolved in different DES-contained aqueous solutions and stored at room temperature. Then, their residual activity as determined at days of 0, 3, 6, 9, 12, 15, 18, 21, 24 and 27, respectively. Based on the enzymatic activity decline curve, the storage stability was evaluated based on storage efficiency defined as the ratio of enzymatic activity after storage to their initial activity.

### 3.5. Investigation of the Optimum Temperature and pH of β-d-Glucosidase in DESs

Four desirable DESs (ChCl/G 2:1; ChCl/G 1:2; ChCl/EG 2:1 and ChCl/Glu 2:1, 20 vol %) were screened based on the enzymatic activity and stability and applied to determine the optimum temperature and pH of β-d-glucosidase. With reference to the above-mentioned method, different water baths at 40, 45, 50, 55, 60, 65 and 70 °C were used; DESs-containing aqueous solutions were prepared to produce the solutions at pH 3.5, 4.0, 5.0, 5.8 and 7.0, respectively. With the maximum value of 100%, the relative activity was determined accurately and calculated from the absorbance, and then the optimum temperature and pH of the enzyme in DESs-containing aqueous solutions were observed intuitively in the diagram.

### 3.6. The Determination of Equilibrium Solubility of Daidzin in Different DESs

The solubility of the substrate daidzin was measured by HPLC analysis according to the saturation dissolved method. The saturated solutions were prepared in a 10 mL centrifuge tube and an excess of solid daidzin was added into each of four DESs (ChCl/G 2:1; ChCl/G 1:2; ChCl/EG 2:1 and ChCl/Glu 2:1) and buffer, while the concentration of DESs were adjusted to 30 vol % according to the preliminary experiments. The solutions were continuously treated with the help of an ultrasonic instrument under the setting temperature for about 3 h to achieve equilibrium and then allow to be stood for another 1 h to obtain a clear saturated solution before sampling. The dissolving temperatures were maintained at 20, 30, 40, 50 and 60 °C, respectively. Then, five samples were extracted from the upper clear saturated solution, diluted 100 times using methanol, and filtered, the filtrate was determined by HPLC, and the processes were as follows:

Carrying out RP-HPLC using a 4.6 mm × 250 mm (5 μm) Zorbax SB-C18 column (Agilent Technologies Industries Co., Ltd.) with the column temperature of 30 °C. The mobile phases were (A) 0.1% acetic acid in water and (B) methanol using a linear gradient elution: 0–4 min, 25% B for isocratic elution; 4–7 min, 25%–32% B; 7–9 min, 32% B for isocratic elution; 9–11 min, 32–50% B; 11–24 min, 50% B for isocratic elution; 24–25 min, 50%–25% B; 25–30 min, 25% B for isocratic elution. UV-detector was set at the wavelength of 254 nm and injection volume was 10 μL for every sample and standard. Equilibrium solubility was calculated from the peak area according to the standard curve. Three parallel tests were conducted at the same time.

### 3.7. General Procedure for the Enzymatic Preparation of Daidzein

First of all, the substrate solution and the enzyme solution were prepared by dissolving an accurately weighed amount of the daidzin and β-d-glucosidase in various DESs-containing aqueous mediums. Subsequently, both of them were incubated under the reaction temperature for 10 min and mixed well. The reaction proceeded by placing the mixture in a constant temperature magnetic stirrer at designated temperatures for a predetermined period of time, while controlling the speed at 200 r/min. At the end of the reaction, the mixture was placed in a water bath at 95 °C for 5 min to quench the reaction. The conversion rate of daidzin was measured by HPLC in accordance with the above-mentioned procedures and calculated from the peak area via the external standard method according to the standard curve before and after reaction as the quantitative parameter.

### 3.8. Optimization of the Enzymatic Reaction Conditions-Box-Behnken Experimental Design

On the basis of the comprehensive and systematical evaluation of enzyme behavior in different DESs, ChCl/EG 2:1 was screened and regarded as the most appropriate medium for enzymatic preparation of daidzein. Response surface methodology (RSM) was used to optimize the enzymatic hydrolysis conditions of daidzin and investigate the correlation between responses and factors. Box-Behnken statistical design (BBD) with 4-factors, 3-levels, and 29 runs was employed to investigate the competitive and interactive effects between the process independent variables by Design-Expert^®^ Version 8.0.6.1 (Stat-Ease, Inc., Minneapolis, MN, USA). According to single factor experiments, four main factors (Reaction time (*X*_1_), Reaction temperature (*X*_2_), pH (*X*_3_) and enzyme loading (*X*_4_)) were chosen and their proper ranges were determined on the basis of the results of initial trials. In accordance with the design, 29 random assort formulations were prepared and characterized for percent hydrolysis of daidzin which were chosen as response parameters (*Y*). Analysis of variance (ANOVA) was used to establish the statistical validation of the polynomial equations generated by Design Expert software. All the responses were fitted to linear, second order and quadratic models, and then evaluated in terms of statistical significance of coefficients and *R*^2^ squared values. The relationship between the response and experimental levels of each factor was established by polynomial equation and the optimum conditions were obtained by 3D response surface and contour plots. The observed response values were compared with the predicted values and the prediction errors (%) were calculated.

### 3.9. Scale-Up Preparation of Daidzein and Medium Reusability

The scale-up preparation of daidzein was implemented under the optimal technological conditions. Meanwhile, we assessed a prospective study to explore the medium reusability. The reaction system was amplified by 10-fold. It was performed in a 100 mL round-bottom flask, the reaction mixture (20 mL) was comprised of a certain amount of the substrate isoflavone 60% daidzin extract and enzyme solution. The reaction was conducted with the same procedure described above, after a period of time, the medium was directly used in the next catalytic cycle. A proper amount of the substrate daidzin was added into the reaction system at a specified time interval and the operation was repeated several times. Through the uninterrupted process of hydrolysis, precipitation was separated out from the system constantly, followed by filtering the solution and washing filter residue with deionized water several times to remove the redundant DESs. Daidzein crude product was obtained after freeze-drying process, and HPLC method was adopted to determine purity of the product. After reaching the preset time, a small amount of mixture was taken and inactivated, then, the conversion yield of daidzein was calculated by HPLC following the above-mentioned method. Moreover, the loss of the enzymatic hydrolysis rate each time and the cycle-index were used to evaluate the operational stability and medium reusability.

### 3.10. Controlled Trials Required by Traditional Hydrolysis Methods

The enzymatic preparation of daidzein in ChCl/EG 2:1, 30 vol % was investigated for the feasibility study and compared to that conducted by acid-catalyzed hydrolysis and enzymatic process in pure phosphate buffer. The acidic hydrolysis was implemented by the traditional heating, and simultaneous ultrasonic/microwave-assisted conditions. The substrate daidzin was dissolved in 3 M concentrated hydrochloric acid solution (the concentration based on the aforementioned optimum conditions), sealed, heated and stirred at 80 °C and 500 rpm. The resulting solution was heated and given ultrasonic processing (40 KHz and 180 W) for 3 h and microwave processing (1000 W) for 0.5 h, followed by dilution with isopyknic methanol at the end of the reaction. Enzymatic hydrolysis of daidzin in pure buffer was executed under the optimum reaction conditions following the same process as described in DESs medium, except for the change of the reaction system. The hydrolysis rate of daidzin with different hydrolytic ways were determined and compared with that previous realized in DESs medium.

## 4. Conclusions

Nowadays, a growing awareness of the chemical process affecting the environment has pushed the research and development of the concept of “green chemistry”. Developing new green reaction solvents is one of the key subjects in green chemistry. As the most pertinent of these solvents, DESs have provided a new versatile and green medium for the biocatalysts reaction due to their particular solvent properties. In this system, some adaptive regularity and enzymatic parameters were determined carefully such that the enzyme activity shows a comparable level and the stability could be efficiently enhanced. In conclusion, we have described the application of ChCl-based DESs as an effective medium for enzymatic preparation of daidzein.

In the process of screening, overall consideration of the influences of DESs for enzyme performance and the substrate solubility, ChCl/EG 2:1 which in the presence of 30 vol % content turns out to be the most effective for carrying out enzymatic preparation of daidzein. The reaction conditions were carefully optimized and determined by RSM combined with Box-Behnken design. Under optimal conditions (reaction temperature 53 °C, pH 5.35, enzyme loading 1.68 U, and reaction time 100.5 min), the maximum conversion of daidzin is up to 97.53%, which agreed well with the RSM prediction. The new process is characterized as mild process conditions, high-efficiency, eco-friendly and easy realization of commercial production. The decent biotransformation demonstrated that β-d-glucosidase in DESs medium has very high reactivity towards hydrolysis. Objectively speaking, DESs medium will hold great promise for the enzymatic reaction, and this work may open new avenues for the large-scale production of daidzein.

## Figures and Tables

**Table 1:** Variables in Box-Behnken design for the preparation of daidzein.

Factors	Actual and Coded Levels Used for the Conditions		
	Low (−1)	Medium (0)	High (+1)
A = Reaction time (min)	30	90	150
B = Temperature (°C)	45	55	65
C = pH value	3.0	5.0	7.0
D = Enzyme loading (U)	0.5	1.5	2.5
Dependent variable (response)		Constrains	
R = Conversion yield (%)		Maximize	

**Table 2:** Analysis of variance (ANOVA) for the quadratic polynomial model for level optimization of enzymatic preparation of daidzein.

Source	Sum of Square	df	Mean Square	*F*-Value	*p*-Value	Significance Level
Model	26,090.77	14	1863.63	62.75	<0.0001	Sig
A-RT	1184.05	1	1184.05	39.87	<0.0001	
B-Temp	3717.12	1	3717.12	125.15	<0.0001	
C-pH	7405.3	1	7405.3	249.32	<0.0001	
D-EL	2745.19	1	2745.19	92.43	<0.0001	
AB	0.16	1	0.16	0.005387	0.9425	
AC	51.12	1	51.12	1.72	0.2106	
AD	0.56	1	0.56	0.019	0.8925	
BC	481.8	1	481.8	16.22	0.0012	
BD	10.56	1	10.56	0.36	0.5605	
CD	128.82	1	128.82	4.34	0.0561	
A^2^	541.98	1	541.98	18.25	0.0008	
B^2^	1769.34	1	1769.34	59.57	<0.0001	
C^2^	9754.11	1	9754.11	328.4	<0.0001	
D^2^	836.1	1	836.1	28.15	<0.0001	
Residual	415.82	14	29.7			
Lack of fit	342.65	10	34.26	1.87	0.2858	Not Sig
Pure Error	73.17	4	18.29			
Cor Total	26,506.59	28				
*R*^2^			0.9843			
Adj *R*^2^			0.9686			
C.V. %			9.74			
Adj precision			23.789			

## Supplementary Materials

Supplementary materials can be accessed at: http://www.mdpi.com/1420-3049/22/1/186/s1.

## References

[1] Watanabe S., Uesugi S., Kikuchi Y. (2002). Isoflavones for prevention of cancer, cardiovascular diseases, gynecological problems and possible immune potentiation. Biomed. Pharmacother..

[2] Levis S., Strickman-Stein N., Doerge D.R., Krischer J. (2010). Design and baseline characteristics of the Soy Phytoestrogens As Replacement Estrogen (SPARE) study—A clinical trial of the effects of soy isoflavones in menopausal women. Contemp. Clin. Trials.

[3] Jing Z., Wei-Jie Y. (2016). Effects of soy protein containing isoflavones in patients with chronic kidney disease: A systematic review and meta-analysis. Clin. Nutr..

[4] Lee M.J., Chung I.-M., Kim H., Jung M.Y. (2015). High resolution LC-ESI-TOF-mass spectrometry method for fast separation, identification, and quantification of 12 isoflavones in soybeans and soybean products. Food Chem..

[5] Day A.J., DuPont M.S., Ridley S., Rhodes M., Rhodes M.J.C., Morgan M.R.A., Williamson G. (1998). Deglycosylation of flavonoid and isoflavonoid glycosides by human small intestine and liver β-glucosidase activity. FEBS Lett..

[6] Setchell K.D.R., Brown N.M., Zimmer-Nechemias L., Brashear W.T., Wolfe B.E., Kirschner A.S., Heubi J.E. (2002). Evidence for lack of absorption of soy isoflavone glycosides in humans, supporting the crucial role of intestinal metabolism for bioavailability. Am. J. Clin. Nutr..

[7] Izumi T., Piskula M.K., Osawa S., Obata A., Tobe K., Saito M., Kataoka S., Kubota Y., Kikuchi M. (2000). Soy isoflavone aglycones are absorbed faster and in higher amounts than their glucosides in humans. J. Nutr..

[8] Iovine B., Iannella M.L., Gasparri F., Giannini V., Monfrecola G., Bevilacqua M.A. (2012). A Comparative Analysis of the Photo-Protective Effects of Soy Isoflavones in Their Aglycone and Glucoside Forms. Int. J. Mol. Sci..

[9] Barnes S. (2010). The Biochemistry, Chemistry and Physiology of the Isoflavones in Soybeans and their Food Products. Lymphat. Res. Biol..

[10] Cassidy A. (2006). Factors affecting the bioavailability of soy isoflavones in humans. J. AOAC Int..

[11] Walsh K.R., Haak S.J., Bohn T., Tian Q., Schwartz S.J., Failla M.L. (2007). Isoflavonoid glucosides are deconjugated and absorbed in the small intestine of human subjects with ileostomies. Am. J. Clin. Nutr..

[12] Liu W., Zhang H.X., Wu Z.L., Wang Y.J., Wang L.J. (2013). Recovery of Isoflavone Aglycones from Soy Whey Wastewater Using Foam Fractionation and Acidic Hydrolysis. J. Agric. Food Chem..

[13] Liu W., Wu Z.L., Wang Y.J., Li R., Yin N.N., Jiang J.X. (2015). Separation of isoflavone aglycones using chitosan microspheres from soy whey wastewater after foam fractionation and acidic hydrolysis. J. Ind. Eng. Chem..

[14] Pei X., Zhao J., Cai P., Sun W., Ren J., Wu Q., Zhang S., Tian C. (2016). Heterologous expression of a GH3 β-glucosidase from Neurospora crassa in Pichia pastoris with high purity and its application in the hydrolysis of soybean isoflavone glycosides. Protein Expr. Purif..

[15] Chang J., Lee Y.-S., Fang S.-J., Park D.-J., Choi Y.-L. (2013). Hydrolysis of isoflavone glycoside by immobilization of β-glucosidase on a chitosan-carbon in two-phase system. Int. J. Biol. Macromol..

[16] Yang S., Wang L., Yan Q., Jiang Z., Li L. (2009). Hydrolysis of soybean isoflavone glycosides by a thermostable β-glucosidase from Paecilomyces thermophila. Food Chem..

[17] Horii K., Adachi T., Matsuda T., Tanaka T., Sahara H., Shibasaki S., Ogino C., Hata Y., Ueda M., Kondo A. (2009). Improvement of isoflavone aglycones production using β-glucosidase secretory produced in recombinant Aspergillus oryzae. J. Mol. Catal. B-Enzym..

[18] Fang W., Yang Y., Zhang X., Yin Q., Zhang X., Wang X., Fang Z., Yazhong X. (2016). Improve ethanol tolerance of β-glucosidase Bgl1A by semi-rational engineering for the hydrolysis of soybean isoflavone glycosides. J. Biotech..

[19] Abbott A.P., Capper G., Davies D.L., Rasheed R.K., Tambyrajah V. (2003). Novel solvent properties of choline chloride/urea mixturesElectronic supplementary information (ESI) available: Spectroscopic data. Chem. Commun..

[20] Abbott A.P., Boothby D., Capper G., Davies D.L., Rasheed R.K. (2004). Deep eutectic solvents formed between choline chloride and carboxylic acids: Versatile alternatives to ionic liquids. J. Am. Chem. Soc..

[21] Garcia G., Aparicio S., Ullah R., Atilhan M. (2015). Deep Eutectic Solvents: Physicochemical Properties and Gas Separation Applications. Energy Fuel.

[22] Troter D.Z., Todorović Z.B., Đokić-Stojanović D.R., Stamenković O.S., Veljković V.B. (2016). Application of ionic liquids and deep eutectic solvents in biodiesel production: A review. Sustain. Energy Rev..

[23] Khandelwal S., Tailor Y.K., Kumar M. (2016). Deep eutectic solvents (DESs) as eco-friendly and sustainable solvent/catalyst systems in organic transformations. J. Mol. Liq..

[24] Craveiro R., Aroso I., Flammia V., Carvalho T., Viciosa M.T., Dionísio M., Barreiros S., Reis R.L., Duarte A.R.C., Paiva A. (2016). Properties and thermal behavior of natural deep eutectic solvents. J. Mol. Liq..

[25] Lindberg D., Revenga M.D.L.F., Widersten M. (2010). Deep eutectic solvents (DESs) are viable cosolvents for enzyme-catalyzed epoxide hydrolysis. J. Biotechnol..

[26] Cvjetko Bubalo M., Curko N., Tomasevic M., Kovacevic Ganic K., Radojcic Redovnikovic I. (2016). Green extraction of grape skin phenolics by using deep eutectic solvents. Food Chem..

[27] Li Z., Lee P.I. (2016). Investigation on drug solubility enhancement using deep eutectic solvents and their derivatives. Int. J. Pharmaceut..

[28] Huang Z.-L., Wu B.-P., Wen Q., Yang T.-X., Yang Z. (2014). Deep eutectic solvents can be viable enzyme activators and stabilizers. J. Chem. Technol. Biotechnol..

[29] Zhao H., Zhang C., Crittle T.D. (2013). Choline-based deep eutectic solvents for enzymatic preparation of biodiesel from soybean oil. J. Mol. Catal. B-Enzym..

[30] Maugeri Z., Leitner W., María P.D.D. (2013). Chymotrypsin-Catalyzed Peptide Synthesis in Deep Eutectic Solvents. Eur. J. Org. Chem..

[31] Gorke J.T., Srienc F., Kazlauskas R.J. (2008). Hydrolase-catalyzed biotransformations in deep eutectic solvents. Chem. Commun..

[32] Gong G.H., Zheng Z.M., Liu H., Wang L., Diao J.S., Wang P., Zhao G.H. (2014). Purification and Characterization of a beta-Glucosidase from Aspergillus niger and Its Application in the Hydrolysis of Geniposide to Genipin. J. Microbiol. Biotechnol..

[33] Wu B.-P., Wen Q., Xu H., Yang Z. (2014). Insights into the impact of deep eutectic solvents on horseradish peroxidase: Activity, stability and structure. J. Mol. Catal. B-Enzym..

[34] Lehmann C., Sibilla F., Maugeri Z., Streit W.R., Domínguez de María P., Martinez R., Schwaneberg U. (2012). Reengineering CelA2 cellulase for hydrolysis in aqueous solutions of deep eutectic solvents and concentrated seawater. Green Chem..

[35] Durand E., Lecomte J., Baréa B., Piombo G., Dubreucq E., Villeneuve P. (2012). Evaluation of deep eutectic solvents as new media for Candida antarctica B lipase catalyzed reactions. Process Biochem..

[36] Durand E., Lecomte J., Baréa B., Dubreucq E., Lortie R., Villeneuve P. (2013). Evaluation of deep eutectic solvent-water binary mixtures for lipase-catalyzed lipophilization of phenolic acids. Green Chem..

[37] Dai Y., Witkamp G.J., Verpoorte R., Choi Y.H. (2015). Tailoring properties of natural deep eutectic solvents with water to facilitate their applications. Food Chem..

[38] Kumar A., Dhar K., Kanwar S.S., Arora P.K. (2016). Lipase catalysis in organic solvents: Advantages and applications. Biol. Proced. Online.

[39] Kotogan A., Kecskemeti A., Szekeres A., Papp T., Chandrasekaran M., Kadaikunnan S., Alharbi N.S., Vagvolgyi C., Tako M. (2016). Characterization of transesterification reactions by Mucoromycotina lipases in non-aqueous media. J. Mol. Catal. B-Enzym..

[40] Weiz G., Braun L., Lopez R., de María P.D., Breccia J.D. (2016). Enzymatic deglycosylation of flavonoids in deep eutectic solvents-aqueous mixtures: Paving the way for sustainable flavonoid chemistry. J. Mol. Catal. B-Enzym..

[41] Yang S.-L., Duan Z.-Q. (2016). Insight into enzymatic synthesis of phosphatidylserine in deep eutectic solvents. Catal. Commun..

[42] Bubalo M.C., Mazur M., Radosevic K., Redovnikovic I.R. (2015). Baker’s yeast-mediated asymmetric reduction of ethyl 3-oxobutanoate in deep eutectic solvents. Process Biochem..

[43] Nie G.J., Zheng Z.M., Jin W., Gong G.H., Wang L. (2012). Development of a tannase biocatalyst based on bio-imprinting for the production of propyl gallate by transesterification in organic media. J. Mol. Catal. B-Enzym..

[44] Wang H., Wang Y., Wang G., Zhang J., Hao H., Yin Q. (2014). Solid-liquid equilibrium of sulbactam in pure solvents and binary solvent mixtures. Fluid Phase Equilib..

[45] Negahdar L., Delidovich I., Palkovits R. (2016). Aqueous-phase hydrolysis of cellulose and hemicelluloses over molecular acidic catalysts: Insights into the kinetics and reaction mechanism. Appl. Cata. B-Environ..

[46] Dikshit R., Tallapragada P. (2015). Partial Purification and Characterization of beta-glucosidase from Monascus sanguineus. Braz. Arch. Biol. Technol..

