# Supplementary material for: Highly Efficient Enzymatic Preparation of Daidzein in Deep Eutectic Solvents
Source: Molecules. 2017 Jan 22;22(1):186. doi: 10.3390/molecules22010186 (PMC6155624; doi:10.3390/molecules22010186)
Supplement: Supplementary file 1 [file molecules-22-00186-s001.pdf]

Supplementary Materials: Highly Efficient Enzymatic Preparation of Daidzein in Deep Eutectic Solvents

Qi-Bin Cheng and Li-Wei Zhang

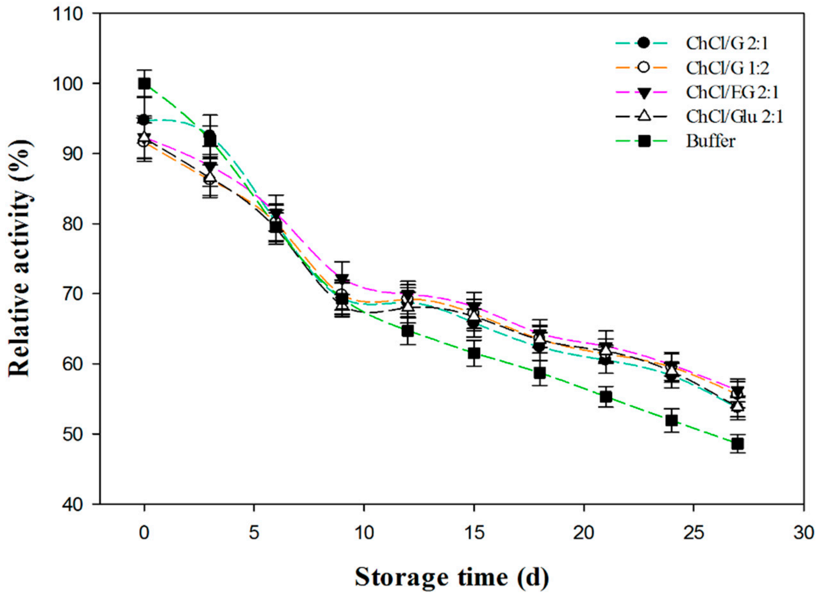

Figure S1. Storage-stability (room temperature) of β-D-glucosidase in phosphate buffer (0.1 M, pH 5.8) containing different DESs (20 vol %).

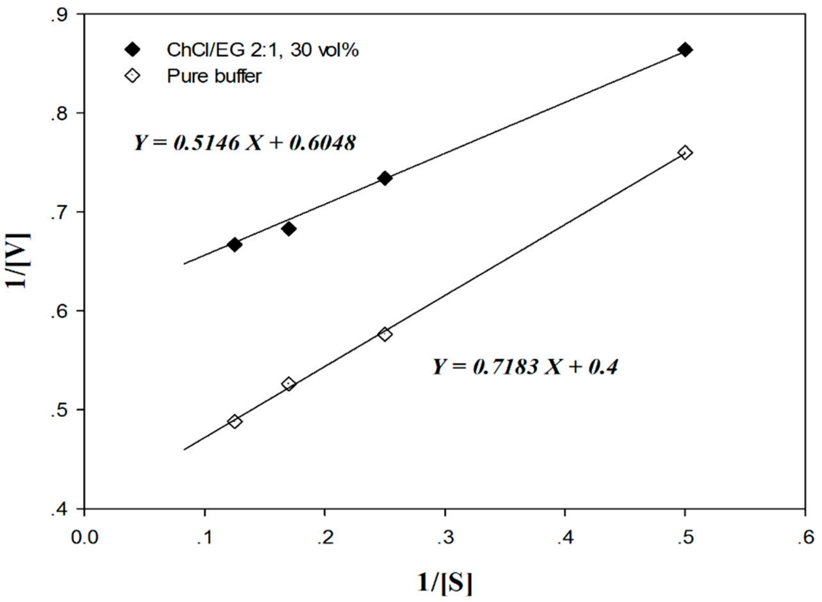

Figure S2. The Lineweaver-Burk plots of β-D-glucosidase in ChCl/EG 2:1 (30 vol %) and phosphate buffer (0.1 M, pH 5.8).

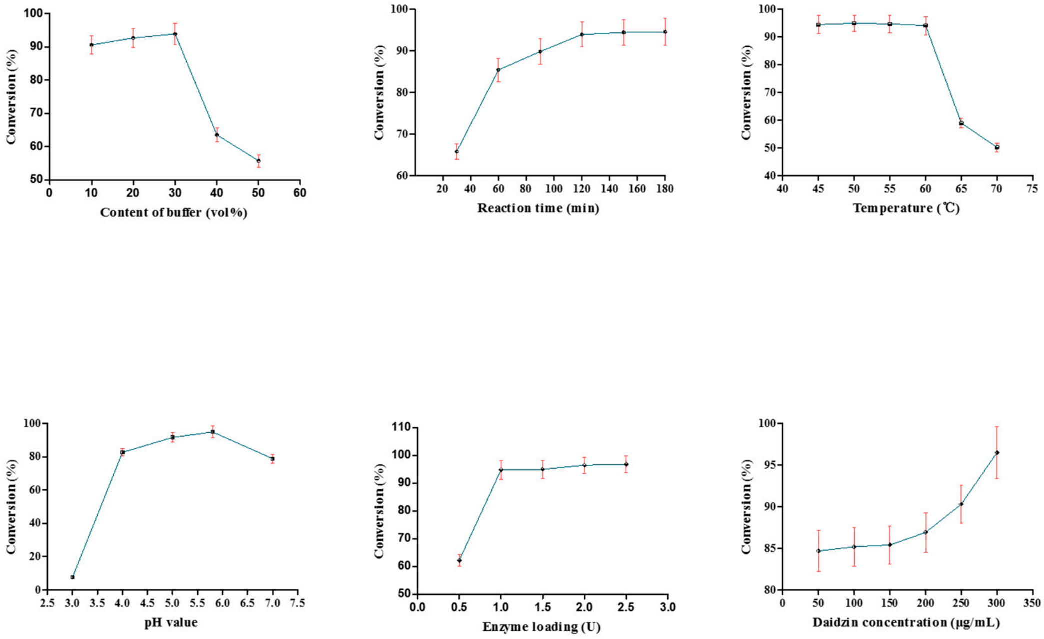

Figure S3. The single-factor test for the enzymatic preparation of daidzein.

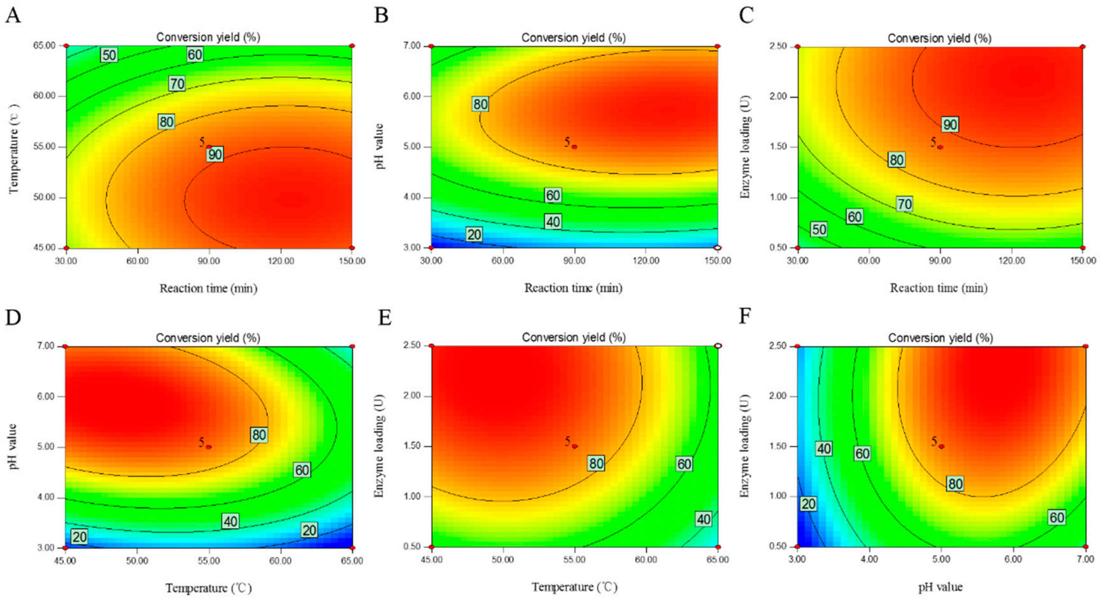

Figure S4. Contour map showing the effects of (**A**) reaction time and temperature; (**B**) reaction time and pH value; (**C**) reaction time and enzyme loading; (**D**) temperature and pH value; (**E**) temperature and enzyme loading; (**F**) pH value and enzyme loading on the conversion yield of daidzein.

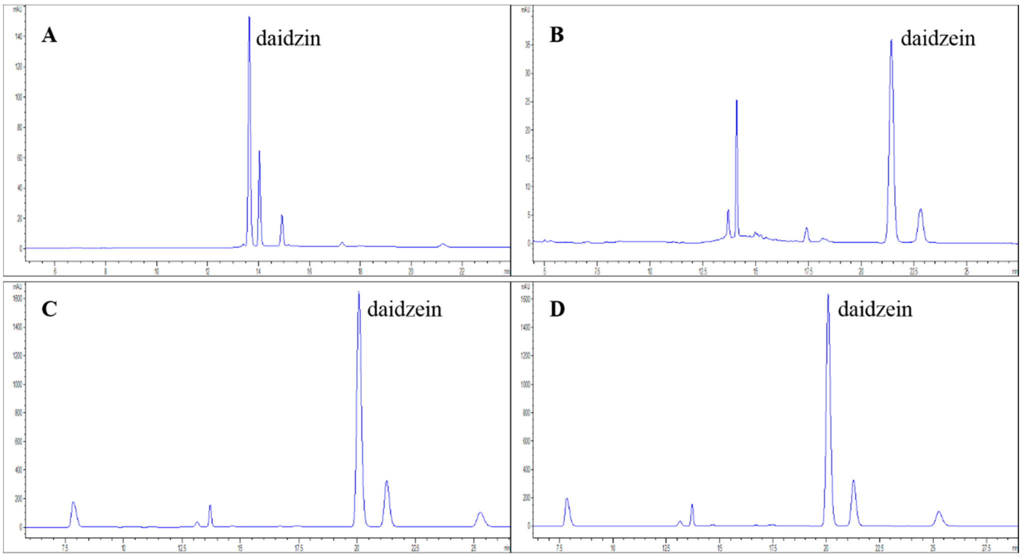

Figure S5. Chromatogram obtained for the reaction before (**A**) and aftern (**B**) and the crude product of daidzein (**C**,**D**).

Table S1. Obseved response in Box-Behnken design (BBD) for the enzymatic preparation of daidzein.

Batch	Preparation of Daidzein				
	Independent Variables				Dependent Variable (Response)
	A (min)	B (°C)	C	D (U)	R = Conversion Yield (%)
1	150	55	5	0.5	57.3
2	90	45	7	1.5	79.5
3	150	55	4	1.5	17.2
4	30	45	5	1.5	69.3
5	90	65	5	2.5	52.6
6	90	55	5	1.5	79.9
7	30	55	5	2.5	76.5
8	90	65	7	1.5	32.5
9	90	55	5	1.5	89.5
10	90	65	3	1.5	7.4
11	30	55	3	1.5	8.1
12	90	55	5	1.5	90.7
13	150	65	5	1.5	52.5
14	90	45	5	2.5	95.8
15	90	45	5	0.5	63.4
16	90	55	3	0.5	6.8
17	90	55	7	2.5	77.8
18	30	65	5	1.5	29.1
19	150	55	5	2.5	96.8
20	30	55	5	0.5	38.5
21	90	55	5	1.5	88.2
22	90	55	3	2.5	18.3
23	90	65	5	0.5	26.7
24	90	55	7	0.5	43.6
25	90	45	3	1.5	10.5
26	150	45	5	1.5	93.5
27	90	55	5	1.5	88.4
28	30	55	7	1.5	54.8
29	150	55	7	1.5	78.2
